# The first report of digenean infecting short mackerel (*Rastrelliger brachysoma*) from Chon Buri Province, the Gulf of Thailand

**DOI:** 10.1007/s00436-024-08308-9

**Published:** 2024-08-07

**Authors:** Chanisara Kaenkaew, Abigail Hui En Chan, Urusa Thaenkham, Napat Ratnarathorn, Sarid Sagulwong, Wallop Pakdee

**Affiliations:** 1 Department of Helminthology, Faculty of Tropical Medicine, Mahidol University, Bangkok, Thailand; 2 Animal Systematics & Molecular Ecology Laboratory and Applied Animal Science Laboratory, Department of Biology, Faculty of Science, Mahidol University, Bangkok, Thailand

**Keywords:** Thailand, Digenea, *Rastrelliger brachysoma*, Molecular identification

## Abstract

*Rastrelliger brachysoma* (Bleeker, 1851), the short mackerel, is a dietary staple and of significant economic demand in Southeast Asia and Thailand. However, the demand for short mackerel has precipitated an overfishing crisis, leading to a depletion of fish stocks. Overfishing, coupled with parasitism, may result in a decline in the population of *R. brachysoma*. Digenetic trematode infection is prevalent in marine fish and has a considerable impact on the overall health of the fish. Here, to identify digenetic trematodes infecting *R. brachysoma*, we aim to determine the identity, prevalence, and intensity of digenean infections in *R. brachysoma* from the Gulf of Thailand. A total of 194 short mackerel were obtained from Chon Buri Province, where digeneans were isolated and identified. The molecular identity of the digeneans was confirmed using the nuclear 28S rRNA gene. Of the 194 short mackerel, 100% were found to be infected with digeneans, comprising of *Lecithocladium*, *Prodistomum*, *Opechona*, and *Aphanurus*. *Lecithocladium* was the most prevalent (98%) and had the highest intensity of infection (37 mean intensity), followed by *Prodistomum* (75% prevalence and 17 mean intensity). Our study thus presents the first evidence of digeneans infecting the economically important short mackerel from the Gulf of Thailand. The high infection rate of digenetic trematodes may have implications on the health of *R. brachysoma*, further driving their population decline. These data underscore the importance of safeguarding fisheries resources in the Gulf of Thailand, and downstream conservation efforts are crucial for evidence-based management decisions to safeguard the long-term sustainability of fish resources.

**Supplementary Information:**

The online version contains supplementary material available at 10.1007/s00436-024-08308-9.

## Introduction

*Rastrelliger brachysoma* (Bleeker, 1851), commonly known as the short mackerel, stands as a linchpin in Southeast Asian economies, and is celebrated for its affordability and rich protein content (Viboonkit et al. 2023). Its ubiquity as a dietary staple, particularly in Thailand, has a profound economic significance, where it enjoys widespread consumer popularity (Indaryanto et al. 2015a; Koolkalya et al. 2020). Despite its ability to spawn throughout the year (Kongseng et al. 2021; Tint et al. 2020), the short mackerel experiences heightened fishing activities from January to March and June to August, signaling its intricate ecological dynamics within the Gulf of Thailand (Tint et al. 2020). This maritime expanse, particularly around Chon Buri Province, emerges as a focal point for short mackerel aggregation, serving as a vital hub for regional export (Tint et al. 2020).

However, the burgeoning demand for short mackerel has precipitated an overfishing crisis, resulting in a stark depletion of stocks in recent years (SEAFDEC 2018). Nevertheless, amidst this ecological challenge, the insidious impact of parasitism on the overall health and resilience of *R. brachysoma* populations may be underestimated. For example, the acanthocephalan *Corynosoma strumosum* was found to be associated with the fertility and population decline of the Caspian sprat (Habibi & Shamsi 2018). Additionally, adult digenetic trematodes, which are prevalent in marine fish, also wield considerable influence on fish health, with their intensity and pathogenicity dictating the severity of the affliction (Abdel-Gaber et al. 2019; Khatoon et al. 2007; Madhavi & Lakshmi 2011; Presswell et al. 2023). Noteworthy among these parasites are hemiurid trematodes such as *Lecithocladium invasor* and *Stomachicola muraenesocis* where infection can compromise the overall fitness and reproductive success of fishes (Keser et al. 2007). *Lecithocladium invasor* was found in the esophagus of acanthurid fishes and can penetrate the esophageal mucosa and induce nodular granulomas (Chambers et al. 2001). Increased host tissue response was also encountered when fish were infected with *Stomachicola muraenesocis* (Nasira et al. 1998).

While prior studies have delved into nematode infections in marine fish from the Gulf of Thailand (Nuchjangreed et al. 2006; Purivirojkul 2009), a conspicuous gap remains regarding investigating digenean, specifically in *R. brachysoma*. To date, there have been no reports of helminths infecting *R. brachysoma* in Thailand. However, parasites have been reported infecting *R. brachysoma* in other Southeast Asian countries. The acanthocephalan belonging to genus *Echinorhynchus* was found in *R. brachysoma* from fish markets in the Philippines (Molina 2021). Additionally, a startlingly high prevalence of the digenean *Lecithocladium angustiovum* (belonging in family Hemiuridae) infection in *R. brachysoma* was discovered in Indonesia, underscoring the urgency of similar investigations within the Gulf of Thailand’s waters (Indaryanto et al. 2015b).

In light of these considerations, our study seeks to bridge this research gap by undertaking an assessment of the prevalence and intensity of digenean trematode infections in short mackerel sourced from the Gulf of Thailand, focusing on Chon Buri Province. We aim to determine the identity of parasitic species infecting *R. brachysoma* and provide preliminary insights into the infection status and identity of digeneans infecting *R. brachysoma* from the Gulf of Thailand. The information obtained may be beneficial to safeguard fisheries resources in the region, ensuring their sustainable management in the future.

## Materials and methods

### Fish sample collection and parasite isolation

A total of 194 short mackerel were obtained from Ang Sila market in Chon Buri Province from May to July 2023. The short mackerels sold at the market were caught off the coast of Ang Sila, from the Gulf of Thailand. All fish were transported back to the Department of Helminthology, Faculty of Tropical Medicine, Bangkok, on ice, where the fish were weighed, measured, and morphologically identified (Supplementary File 1).

The short mackerels were dissected carefully within 48 h of collection, and the internal organs of the gastrointestinal tract (stomach, intestine, and pyloric caeca) were thoroughly examined under a stereomicroscope for the presence of digeneans. The isolated digeneans were sorted to the genus level based on their morphological characters according to the Keys to Trematoda (Bray et al. 2008; Gibson et al. 2002; Jones et al. 2005), and they were then counted and kept in 70% ethanol at − 20 °C for downstream molecular identification.

### Data analysis

The data obtained (Supplementary File 1) were incorporated into Microsoft Excel, and the proportion, prevalence, and mean intensity were calculated. The formulas are provided as follows (Bush et al. 1997):

$$\text{Proportion}=\frac{\text{Total number of specific parasite}}{\text{Total number of parasites}} \times 100$$

$$\text{Prevalance}= \frac{\text{Total number of fish infected with specific parasite}}{\text{Total number of fish examined}} \times 100$$

$$\text{Mean intensity}= \frac{\text{Total number of specific parasite}}{\text{Number of infected fish}}$$

### Molecular confirmation

Representative specimens were individually placed into 1.5-ml microcentrifuge tubes and washed thoroughly with sterile distilled water. Total genomic DNA was isolated from each specimen using the Geneaid genomic DNA mini kit (Geneaid Biotech Ltd., Taipei, Taiwan) following the manufacturer’s recommendations.

Polymerase chain reaction (PCR) was conducted in a T100™ thermocycler (Bio-Rad, California, USA) to amplify the partial nuclear 28S ribosomal RNA (rRNA) gene. The primers used (28S-F: 5′-AAGCATATCACTAAGCGG-3′ and 28S-R: 5′-GCTATCCTGAGGGAAACTTCG-3′) and thermocycling conditions applied follow Curran et al. (2011) (Curran et al. 2011). The 1200 base pairs (bp) amplicons were visualized on a 1% agarose gel stained with SYBR™ Safe (Thermo Fisher Scientific, MA, USA). PCR amplicons were sent for Barcode Taq sequencing (Celemics, Seoul, South Korea) performed by a commercial company.

Following sequencing, electropherograms were manually checked using BioEdit 7.0 and aligned using ClustalX 2.1 together with reference sequences obtained from the NCBI database (Thompson et al. 2002; Hall 1999). The reference sequences used are in Supplementary File 2. The aligned sequences were checked in BioEdit 7.0, and phylogenetic analysis using the neighbour-joining (NJ) and maximum likelihood (ML) method was performed with MEGA X (Kumar et al. 2018). The best-fit nucleotide substitution model with 1000 bootstrap iterations for tree topology was selected for the ML method. *Echinostoma* was used as outgroups to root the phylogenetic trees. The phylogenetic trees were visualized and labeled with FigTree 1.3.1 (Rambuat 2010).

## Results

### Infection status of *Rastrelliger brachysoma* with digeneans

Of the 194 *R. brachysoma* obtained, 100% of them were found to be infected with digeneans. Four genera, consisting of *Lecithocladium*, *Aphanurus*, *Prodistomum*, and *Opechona*, were found. Of the four genera, infection with *Lecithocladium* was the most prevalent (98%), followed by *Prodistomum* (75%), *Opechona* (34%), then *Aphanurus* (20%). In terms of the mean intensity of infection, *Lecithocladium* was the highest at 37 (range from 1 to 344 individuals per infected fish), followed by *Prodistomum*, *Aphanurus*, and *Opechona*. Among the four genera, *Lecithocladium* comprised the highest proportion (71%), followed by *Prodistomum* (26%), *Opechona* (1.7%), and *Aphanurus* (1.3%). *Lecithocladium* was found primarily in the stomach of the short mackerel, while *Prodistomum* was found in the pyloric caeca and intestine. Table 1 presents the prevalence, mean intensity, and proportion observed for the four genera.

**Table 1:** Prevalence, mean intensity, and proportion of digenean infecting *R. brachysoma*

Genus	Number of infected *R. brachysoma*	Number of individual digenean isolated	Prevalence (%)	Mean intensity (minimum–maximum)^a^	Proportion (%)
*Lecithocladium*	192	7262	98	37 (1**–**344)	71.0
*Prodistomum*	147	2613	75	17 (1**–**122)	26.0
*Opechona*	67	183	34	2 (1**–**36)	1.7
*Aphanurus*	39	134	20	3 (1**–**16)	1.3
Total	194	10,192			

^a^The minimum to maximum number of individual parasite isolated from each infected fish

**Fig. 1:**
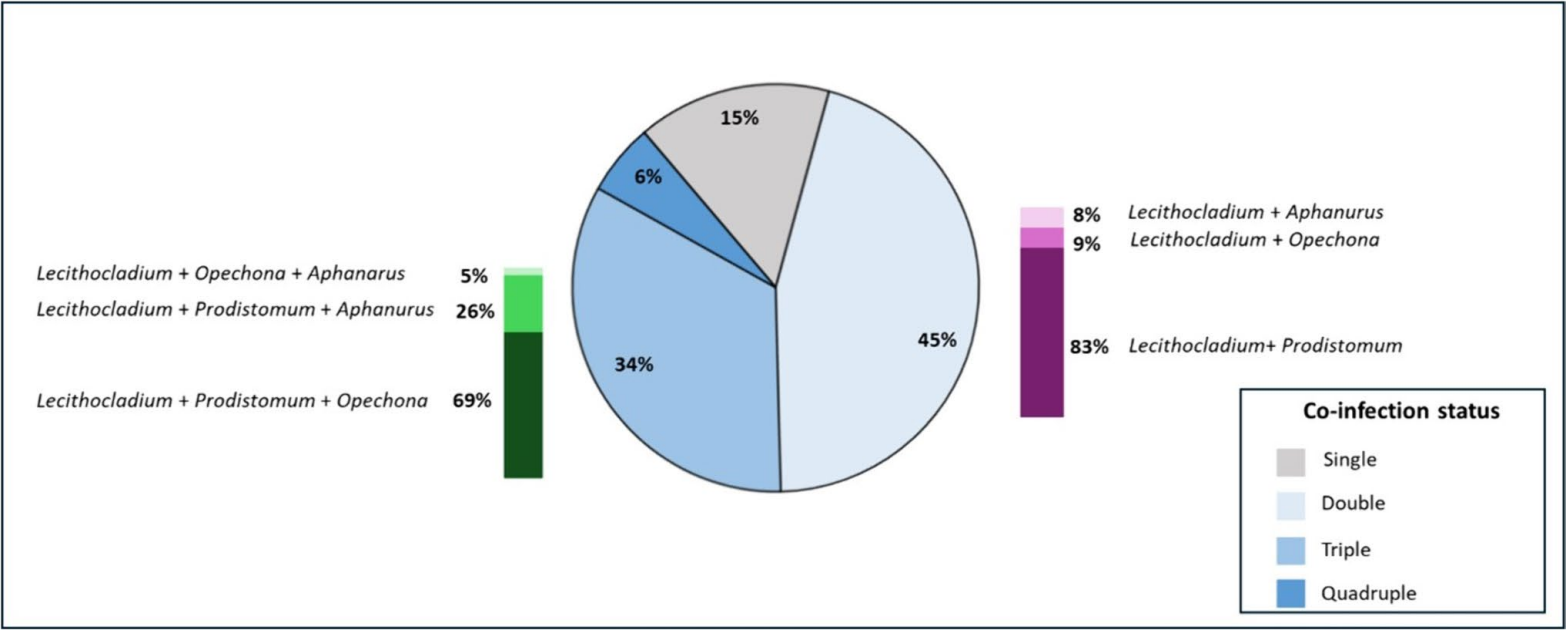
Proportion of infected *R. brachysoma* with single and multiple infection

Digenetic infection with more than one genus per fish was also present, where co-infection with two and three genera was common, comprising 45% and 34% of the infected short mackerels, respectively (Fig. 1). Fifteen percent of the infected mackerels had a single infection, while 6% were co-infected with all four genera. Co-infection by *Lecithocladium* and *Prodistomum* was the most common of those infected with two genera.

The pie chart depicts the co-infection status of *R. brachysoma* presented as percentage of fish with single, double, triple, or quadruple infection with the four digenetic genera. The purple and green stacked bar chart depicts the combination of digenetic genera infecting in *R. brachysoma* for double and triple infection, respectively.

### Molecular confirmation using the nuclear 28S rRNA gene

Using the nuclear 28S rRNA gene, molecular results revealed congruence with the initial morphological identity of the four genera of digeneans isolated from *R. brachysoma*.

**Fig. 2:**
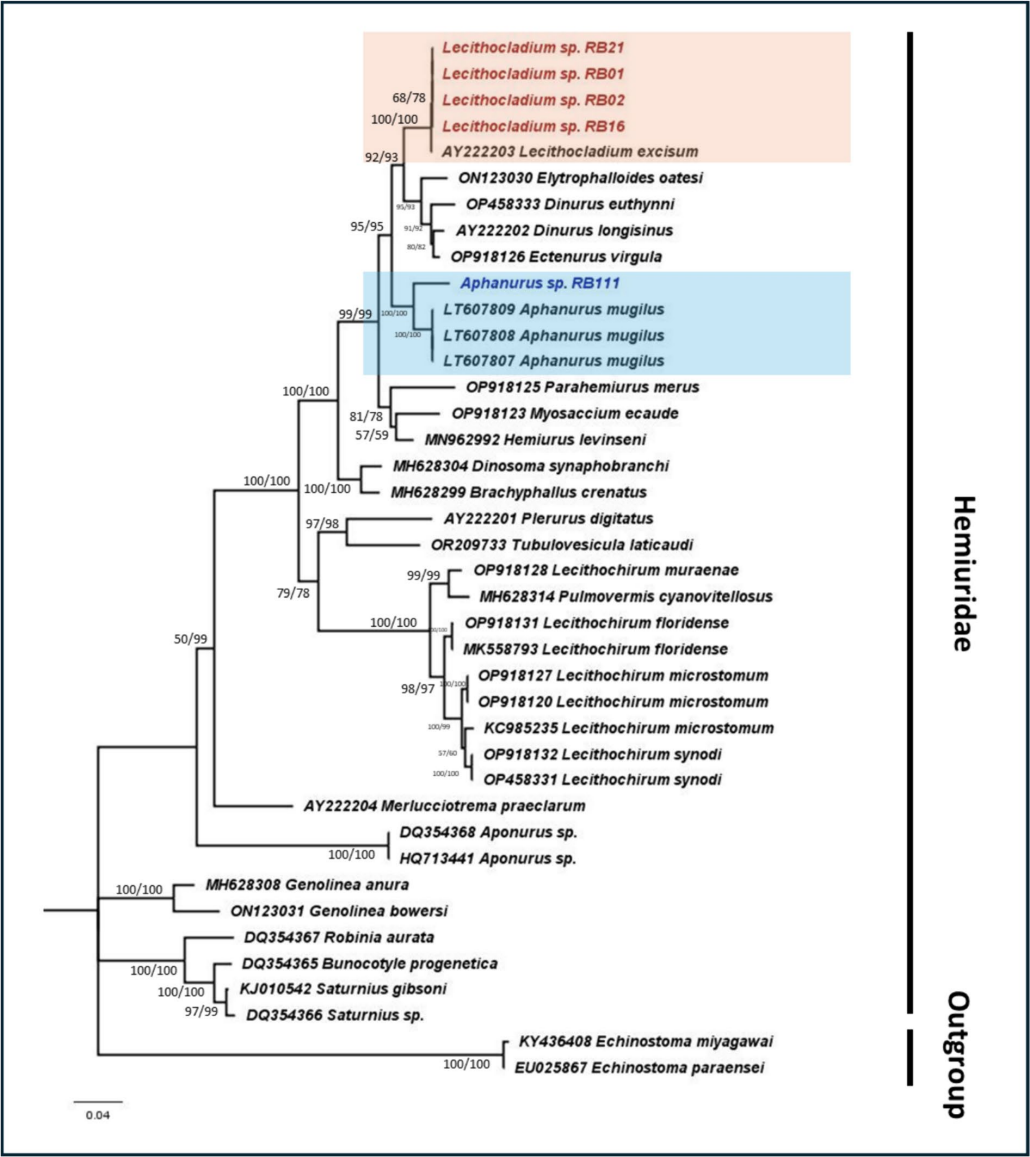
Maximum likelihood (GTR G + I) phylogeny of family Hemiuridae based on the partial nuclear 28S rRNA gene sequences as genetic marker

The molecular phylogeny of the family Hemiuridae, consisting of *Lecithocladium* and *Aphanurus*, revealed a monophyletic clade for *Lecithocladium* (our *Lecithocladium* specimens and *Lecithocladium excisum*) with strong bootstrap support. Genetic distances between our *Lecithocladium* specimens and *L. excisum* were 0.009, while no intraspecies genetic variation was observed within our specimens. Similarly, our *Aphanurus* specimens formed a monophyletic clade with *Aphanurus mugilus*, with a genetic distance of 0.041. Figure 2 presents the phylogeny of family Hemiuridae.

**Fig. 3:**
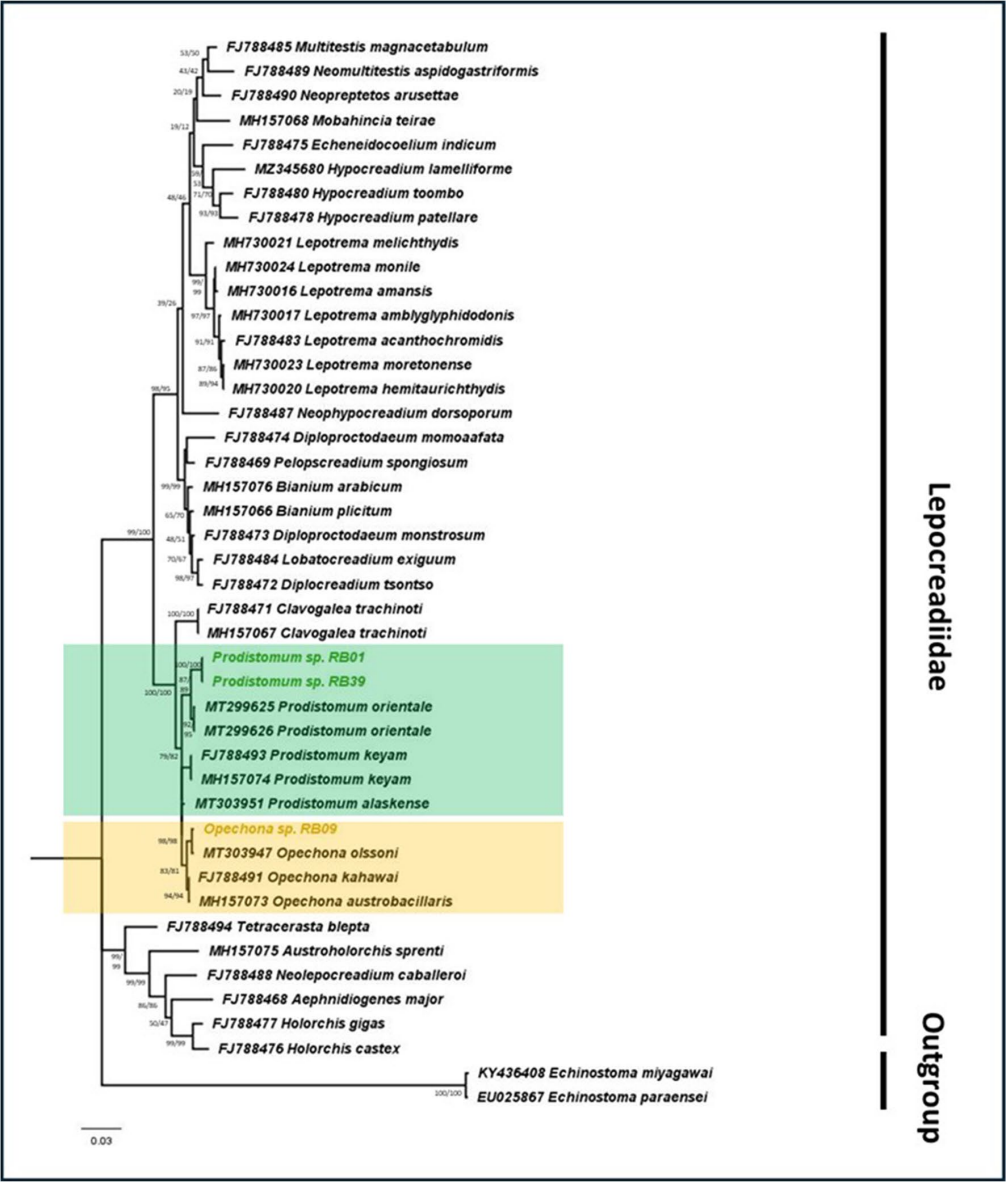
Maximum likelihood (GTR G + I) phylogeny of family Lepocreadiidae based on the partial nuclear 28S rRNA gene sequences as genetic marker

The molecular phylogeny of the family Lepocreadiidae, consisting of *Prodistomum* and *Opechona*, also supported the congruence of their morphological identity (Fig. 3). The phylogeny revealed a close relationship between our *Prodistomum* specimens and reference *Prodistomum orientale*, in which this monophyletic group formed a sister clade to *Prodistomum keyam*. The genetic distance between our *Prodistomum* specimens and *P. orientale* was 0.009, and no intraspecies variation was observed among our *Prodistomum* specimens. Similarly, our *Opechona* specimen clustered together with *Opechona olssoni*, where the genus *Opechona* was revealed to be monophyletic. The genetic distance between our *Opechona* specimen and *O. olssoni* was 0.001. Additionally, the sister group relationship between the two genera (*Prodistomum* and *Opechona*) was observed with the 28S rRNA gene phylogeny.

The numbers at the nodes indicate bootstrap (BS) support obtained through 1000 replications (ML/NJ). NCBI sequences are shown with their accession numbers. The representative sequences for *Lecithocladium* and *Aphanurus* are highlighted in “red” and “blue,” respectively.

The numbers at the nodes indicate bootstrap (BS) support obtained through 1000 replications (ML/NJ). NCBI sequences are shown with their accession numbers. The representative sequences for *Prodistomum* and *Opechona* are highlighted in “green” and “yellow,” respectively.

## Discussion

We confirmed the presence of adult digeneans belonging to four distinct genera, highlighting the prevalence and intensity of infection within this fish population. Additionally, all the short mackerel obtained were infected with digeneans. The identity of these digeneans and their respective prevalence provides a crucial foundation regarding the infection status to understand parasitic infections in *R. brachysoma*, underlining the need for targeted research and management strategies to mitigate their impact.

Among the four genera identified, *Lecithocladium* emerged as the most prevalent, exhibiting the highest intensity of infection. This finding aligns with previous research conducted in Thailand, where *Lecithocladium cristatum* was isolated and identified from the black pomfret fish (*Parastromateus niger*) in markets in Bangkok (Oopkaew et al. 2020). The high prevalence of *Lecithocladium* (72%) in another fish species demonstrates the potential for cross-species transmission of digenean parasites within marine ecosystems, posing a significant threat to the health and sustainability of multiple fish species. While the presence of adult digeneans in marine fish does not directly impact human health, parasitic infections can have cascading effects on the health, quality, and market value of the fish caught. Moreover, as fishes caught from the Gulf of Thailand are transported to markets in various provinces in Thailand, the knowledge of parasitic infection in *R. brachysoma* may have an impact on consumer preferences, further affecting their market value of *R. brachysoma*. Thus, effective fisheries management and disease surveillance programs are crucial to mitigate the impact of parasitic infection in fishes (Cribb et al. 2002; Khatoon et al. 2007).

Furthermore, the pathological effects of *Lecithocladium* infection on the gastrointestinal tract of marine fish have been documented globally. Pathological changes, including severe inflammation, tissue atrophy, and lysis, have been observed in fish species such as *Naso vlamingii* infected with *L. invasor* and *Lecithocladium chingi* (Chambers et al. 2001). Similarly, observations of gastrointestinal tract damage due to *Lecithocladium* and *Bucephalus* infection in the orange-spotted trevally (*Carangoides bajad*) from the Red Sea highlight the broad geographic distribution and ecological impact of these parasitic infections (Bakhraibah & Bin Dohaish 2023). These findings underscore the urgent need for comprehensive surveillance and management efforts to mitigate the spread and impact of digenean parasites on marine ecosystems worldwide.

Given the high value and economic importance of short mackerel for countries bordering the Gulf of Thailand, collaborative efforts have been made to mitigate the decline of the short mackerel population (Tint et al. 2020). These efforts include the transboundary promotion of collaborative fishing management and data collection regarding the short mackerel population. Also, refugia sites were demarcated in Trat Province, aiming to mitigate the impact of short mackerel overfishing (Munprasit et al. 2022). By providing the first evidence of digenetic trematode infection in *R. brachysoma*, we underscore the importance of sustainably protecting this valuable fish species, as population decline may be further exacerbated by parasitic infection and overfishing. Additionally, the partial 28S rRNA gene sequences of the four genera are provided, which may facilitate future research in enhancing genetic information available for digenetic trematodes in marine fishes.

## Conclusion

In conclusion, our study presents the first evidence of digeneans infecting economically important short mackerel from the Gulf of Thailand in Chon Buri Province. This information holds significance for future management strategies to protect fish resources in Thailand. However, as only one genetic marker was utilized in this study, incorporating additional genetic markers from different loci and genetic markers with better taxonomic resolution may aid in species-level identification of isolated digeneans for future investigations. Furthermore, future sampling of short mackerels from various localities along the Gulf of Thailand has the potential to expand our understanding of the parasitic infection status of *R. brachysoma* in Thailand. These efforts are crucial for informing evidence-based management decisions and safeguarding the long-term sustainability of marine ecosystems and fisheries resources.

## Supplementary Information

Below is the link to the electronic supplementary material.

Supplementary file1 (XLSX 27 KB)

Supplementary file2 (DOCX 22 KB)

## Data Availability

All data generated during this study are included in the published article and its additional files. The newly generated sequences were deposited in the GenBank database under the accession numbers PP768936 – PP768943.
