# Supplementary material for: The first report of digenean infecting short mackerel (Rastrelliger brachysoma) from Chon Buri Province, the Gulf of Thailand
Source: Parasitol Res. 2024 Aug 7;123(8):294. doi: 10.1007/s00436-024-08308-9 (PMC11303497; doi:10.1007/s00436-024-08308-9)
Supplement: Supplementary file 2 — Supplementary file2 (DOCX 22 KB) [file 436_2024_8308_MOESM2_ESM.docx]

**Supplementary file 2: List of sequences used for phylogenetic analysis**

| **Family** | **Species** | **NCBI accession number** |
| --- | --- | --- |
| Hemiuridae | *Lecithocladium excisum* | AY222203 |
|  | *Lecithocladium* sp. RB01 | PP768936 (This study) |
|  | *Lecithocladium* sp. RB02 | PP768937 (This study) |
|  | *Leciithocladium* sp. RB16 | PP768938 (This study) |
|  | *Lecithocladium* sp. RB21 | PP768939 (This study) |
|  | *Elytrophalloides oatesi* | ON123030 |
|  | *Dinurus euthynni* | OP458333 |
|  | *Dinurus longisinus* | AY222202 |
|  | *Ectenurus virgula* | OP918126 |
|  | *Aphanurus mugilus* | LT607809 |
|  | *Aphanurus mugilus* | LT607808 |
|  | *Aphanurus mugilus* | LT607807 |
|  | *Aphanurus* sp. RB111 | PP768940 (This study) |
|  | *Parahemiurus merus* | OP918125 |
|  | *Myosaccium ecaude* | OP918123 |
|  | *Hemiurus levinseni* | MN962992 |
|  | *Dinosoma synaphobranchi* | MH628304 |
|  | *Brachyphallus crenatus* | MH628299 |
|  | *Plerurus digitatus* | AY222201 |
|  | *Tubulovesicula laticaudi* | OR209733 |
|  | *Lecithochirum muraenae* | OP918128 |
|  | *Pulmovermis cyanovitellosus* | MH628314 |
|  | *Lecithochirum floridense* | OP918131 |
|  | *Lecithochirum floridense* | MK558793 |
|  | *Lecithochirum microstomum* | OP918127 |
|  | *Lecithochirum microstomum* | OP918120 |
|  | *Lecithochirum microstomum* | KC985235 |
|  | *Lecithochirum synodi* | OP918132 |
|  | *Lecithochirum synodi* | OP458331 |
|  | *Merlucciotrema praeclarum* | AY222204 |
|  | *Aponurus* sp. | DQ354368 |
|  | *Aponurus* sp. | HQ713441 |
|  | *Genolinea anura* | MH628308 |
|  | *Genolinea bowersi* | ON123031 |
|  | *Robinia aurata* | DQ354367 |
|  | *Bunocotyle progenetica* | DQ354365 |
|  | *Saturnius gibsoni* | KJ010542 |
|  | *Saturnius* sp. | DQ354366 |
| Lepocreadiidae | *Prodistomum orientale* | MT299625 |
|  | *Prodistomum orientale* | MT299626 |
|  | *Prodistomum* sp. RB01 | PP768941 (This study) |
|  | *Prodistomum* sp. RB39 | PP768942 (This study) |
|  | *Prodistomum keyam* | FJ788493 |
|  | *Prodistomum keyam* | MH157074 |
|  | *Prodistomum alaskense* | MT303951 |
|  | *Opechona olssoni* | MT303947 |
|  | *Opechona kahawai* | FJ788491 |
|  | *Opechona austrobacillaris* | MH157073 |
|  | *Opechona* sp. RB09 | PP768943 (This study) |
|  | *Tetracerasta blepta* | FJ788494 |
|  | *Austroholorchis sprenti* | MH157075 |
|  | *Neolepocreadium caballeroi* | FJ788488 |
|  | *Aephnidiogenes major* | FJ788468 |
|  | *Holorchis gigas* | FJ788477 |
|  | *Holorchis castex* | FJ788476 |
|  | *Multitestis magnacetabulum* | FJ788485 |
|  | *Neomultitestis aspidogastriformis* | FJ788489 |
|  | *Neopreptetos arusettae* | FJ788490 |
|  | *Mobahincia teirae* | MH157068 |
|  | *Echeneidocoelium indicum* | FJ788475 |
|  | *Hypocreadium lameliiforme* | MZ345680 |
|  | *Hypocreadium toombo* | FJ788480 |
|  | *Hypocreadium patellare* | FJ788478 |
|  | *Lepotrema melichtydis* | MH730021 |
|  | *Lepotrema monile* | MH730024 |
|  | *Lepotrema amansis* | MH730016 |
|  | *Lepotrema amblyglyphidodonis* | MH730017 |
|  | *Lepotrema acanthochromidis* | FJ788483 |
|  | *Lepotrema moretonense* | MH730023 |
|  | *Lepotrema hemitaurichthydis* | MH730020 |
|  | *Neophypocreadium dorsoporum* | FJ788487 |
|  | *Diploproctodaeum momoaafata* | FJ788474 |
|  | *Pelopscreadium spongiosum* | FJ788469 |
|  | *Blanium arabicum* | MH157076 |
|  | *Blanium plictum* | MH157066 |
|  | *Diploproctodaeum monstrosum* | FJ788473 |
|  | *Lobatocreadium exiguum* | FJ788484 |
|  | *Diplocreadium tsontso* | FJ788472 |
|  | *Clavogalea trachinoti* | FJ788472 |
|  | *Clavogalea trachinoti* | MH157067 |
| Outgroups | *Echinostoma miyagawai* | KY436408 |
|  | *Echinostoma paraensei* | EU025867 |
